# Acinar-to-Ductal Metaplasia (ADM): On the Road to Pancreatic Intraepithelial Neoplasia (PanIN) and Pancreatic Cancer

**DOI:** 10.3390/ijms24129946

**Published:** 2023-06-09

**Authors:** Louis Marstrand-Daucé, Diane Lorenzo, Anaïs Chassac, Pascal Nicole, Anne Couvelard, Cécile Haumaitre

**Affiliations:** 1 INSERM UMR1149, Inflammation Research Center (CRI), Université Paris Cité, 75018 Paris, France; louis.marstrand-dauce@etu.u-paris.fr (L.M.-D.); diane.lorenzo@inserm.fr (D.L.); anais.chassac@aphp.fr (A.C.); pascal.nicole@inserm.fr (P.N.); anne.couvelard@aphp.fr (A.C.); 2 Department of Pathology, Bichat Hospital, Université Paris Cité, 75018 Paris, France

**Keywords:** acinar-to-ductal metaplasia (ADM), pancreatic intraepithelial neoplasia (PanIN), pancreatic ductal adenocarcinoma (PDAC), pancreatitis, tumorigenesis, trans-differentiation, regeneration, proliferation, inflammation

## Abstract

Adult pancreatic acinar cells show high plasticity allowing them to change in their differentiation commitment. Pancreatic acinar-to-ductal metaplasia (ADM) is a cellular process in which the differentiated pancreatic acinar cells transform into duct-like cells. This process can occur as a result of cellular injury or inflammation in the pancreas. While ADM is a reversible process allowing pancreatic acinar regeneration, persistent inflammation or injury can lead to the development of pancreatic intraepithelial neoplasia (PanIN), which is a common precancerous lesion that precedes pancreatic ductal adenocarcinoma (PDAC). Several factors can contribute to the development of ADM and PanIN, including environmental factors such as obesity, chronic inflammation and genetic mutations. ADM is driven by extrinsic and intrinsic signaling. Here, we review the current knowledge on the cellular and molecular biology of ADM. Understanding the cellular and molecular mechanisms underlying ADM is critical for the development of new therapeutic strategies for pancreatitis and PDAC. Identifying the intermediate states and key molecules that regulate ADM initiation, maintenance and progression may help the development of novel preventive strategies for PDAC.

## 1. Introduction

The pancreas composed of endocrine and exocrine components is an important organ for the regulation of food digestion and blood glucose balance. In human or mouse pancreas, exocrine cells account for more than 90% of the organ. Acinar cells, the main component of exocrine tissue, are polarized epithelial cells that are responsible for the production of digestive enzymes, including amylase, protease, lipase and trypsin. Terminally differentiated, secretory acinar cells are normally post-mitotic and store zymogen granules filled with these enzymes that are secreted by exocytosis. Digestive enzymes are transported in a network of ducts that discharge pancreatic juices into the duodenum. Ductal cells are responsible for producing and secreting bicarbonate ions that neutralize the acidic contents of the stomach as they enter the small intestine.

**Figure 1:**
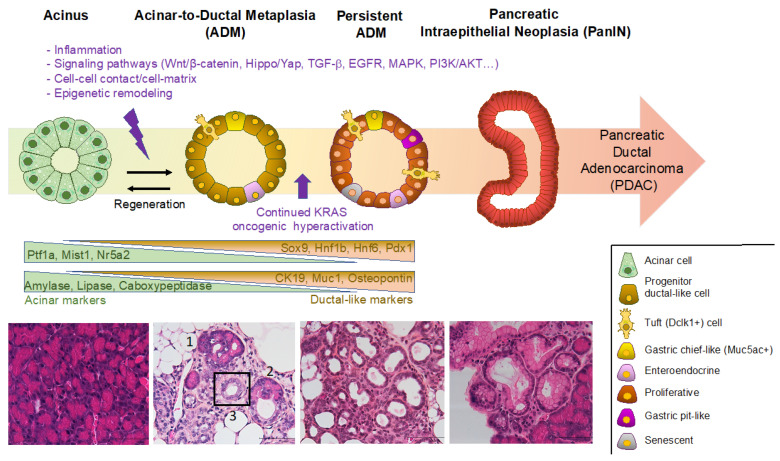
Model of acinar-to-ductal metaplasia (ADM) initiation and progression to pancreatic intraepithelial neoplasia (PanIN). ADM formation is illustrated and triggering signals of ADM as well as changes in expression markers are shown. ADM is a reversible process that enables the regeneration of pancreatic acinar tissue. However, if there is sustained oncogenic Kras activation, ADM can become persistent, and acinar cell reprogramming can lead to the formation of PanIN and progression to pancreatic ductal adenocarcinoma (PDAC). Acinar cells exhibit substantial plasticity and heterogeneity while undergoing metaplasia. The corresponding histological stages in murine tissue, including normal acinar cells, ADM and PanIN, are shown with Haematoxylin/Eosin staining. Transition during ADM is observed in the second histological panel, where the lumen of acini enlarges (1), an intermediate structure is formed (2), and a ductal-like structure of ADM is framed (3). Scale bar: 100 μm. (Images adapted with permission from Ref. [3], Copyright 2023, Elsevier).

Pancreatic tissue homeostasis is a regular process of cellular renewal. The homeostatic balance in pancreas is critical for its normal functions and is disturbed during tissue injury, inflammation and tumorigenesis. Acinar-to-ductal metaplasia (ADM) is a process that corresponds to pancreatic acinar cells dedifferentiating into ductal-like cells. During ADM, the acinar cells lose their characteristic shape and function and adopt a ductal-like cell morphology. The process involves changes in the expression of genes that control cell differentiation, proliferation, and survival. It shows the ability of acinar cells to adapt to the genetic and environmental pressure. However, the exocrine cellular plasticity within the pancreas is exploited in tumorigenesis, with metaplastic, dedifferentiation and trans-differentiation processes leading to the development of pancreatic intraepithelial neoplasia (PanIN). The whole process is illustrated in Figure 1. 

Pancreatic Ductal Adenocarcinoma (PDAC) is the fourth leading cause of cancer-related deaths worldwide. The projection of incidence in 2030 indicates that PDAC will become the second most prevalent cause of cancer-related death [1]. As PDAC is often diagnosed at an advanced stage, the 5-year survival rate is very low, less than 10%. Pancreatic intraepithelial neoplasia (PanIN) refers to the most frequent PDAC precursor lesions. These microscopic noninvasive epithelial preneoplastic lesions that are not detectable in humans by radiological examination exhibit varying mucin levels and degrees of cytologic atypia [2]. With oncogenic genetic insults and/or sustained environmental stress, ADM can lead to PanIN, preceding PDAC. 

Here, we provide a review on the definition of ADM and the transcription factors involved, the signaling pathways triggering ADM, and the progression of ADM to PanIN. 

## 2. Definition of Acinar-to-Ductal Metaplasia (ADM) and Transcription Factors Involved

Metaplasia is a reversible change in which one differentiated cell type is replaced by another cell type. This process can occur in response to various stimuli, such as chronic inflammation or cellular injury. The metaplasia of pancreatic acinar cells manifests their ability to adapt to the genetic and environmental pressure they encounter. Many studies have shown the crucial role of acinar cells in post-injury pancreatic regeneration [4,5,6]. During this process, acinar cells undergo ADM—which is the traditionally used terminology—by losing their mature and functional characteristics as well as undergoing a morphological and transcriptional transformation into ductal-like cells with embryonic progenitor properties [7,8,9]. Like pancreatic progenitor cells, ADM cells are proliferative, whereas mature acinar and ductal cells are largely mitotically quiescent. ADM cannot be considered as a trans-differentiation event corresponding to direct conversion from acinar to ductal cells, as acinar cells dedifferentiate into an embryonic progenitor-like phenotype and differentiate into duct-like cells. The terminology of metaplasia, trans-differentiation and dedifferentiation is a matter of debate [10]. Recently, the term paligenosis, described as the biological process of converting a mature cell into a regenerative cell, was also proposed [11]. In the context of ADM, acinar cell reprogramming can englobe the different terminologies. ADM appears to be a protective mechanism that temporarily reduces extensive tissue damage caused by excessive pancreatic secretion of digestive enzymes. The damaged tissue may return to normal if the stimulus causing metaplasia is removed. Metaplasia, however, can progress to dysplasia and tumors if the stimuli that promote it persist. 

The process is evolutionarily conserved as it happens in rodents (references below) and humans [12,13,14,15,16]. ADM was demonstrated with the use of genetically engineered mouse models (GEMMs) (references below) and in vitro in 3D cell culture with mouse and human primary acinar cells [17].

Morphologically, zymogene granules are gradually lost by acinar cells, which show a reduced apical cytoplasm while the lumen of the acini increases. Acinar cells lose polarity and acquire a cuboidal–columnar morphology resembling ductal precursors of the embryonic pancreas. During this process, ADM structures composed of both acinar and duct-like cells can be observed. In the later stages, ADM structures are composed only of duct-like cells, making them difficult to distinguish from pancreatic branched ducts.

The shift from acinar cells to ductal-like cells relies on a down-regulation of acinar gene expression (see Figure 1). The expression of acinar-specific transcription factors including Ptf1a, Mist1 and Nr5a2 is reduced, as well as the expression of digestive enzymes such as carboxypeptidase and amylase, leading to a gradual loss of digestive enzyme synthesis and secretory functions. During pancreas development, Ptf1a is required for the maintenance of multipotent progenitor cells (MPC) [18,19]. After E12.5, Ptf1a expression is restricted to the tip of the pancreatic epithelium adopting an acinar fate, while the cells in the trunk become restricted to a ductal or endocrine fate [20]. Ptf1a is required for maintenance of acinar cell identity by forming a complex network regulating acinar cell-specific digestive enzyme genes [7,21,22,23]. Loss of Ptf1a in acinar cells is sufficient to induce ADM and potentiate inflammation [24,25]. Mist1, restricted to acinar cells throughout development and in adult tissue [26], is a key regulator of acinar cell function, proliferation and identity maintenance [26,27]. Mist1 plays a protective role in ADM. Inhibition of Mist1 aggravated ADM [28,29,30], whereas forced expression of Mist1 significantly attenuated ADM [31]. Nr5a2, a member of the nuclear receptor family of ligand-activated transcription factors, maintains the secretory functions of acinar cells and is a key regulator of acinar cell plasticity. Loss of Nr5a2 accelerates the ADM process, and it was shown that Nr5a2 is required for maintenance of acinar identity and re-establishment of acinar fate during regeneration [32].

**Figure 2:**
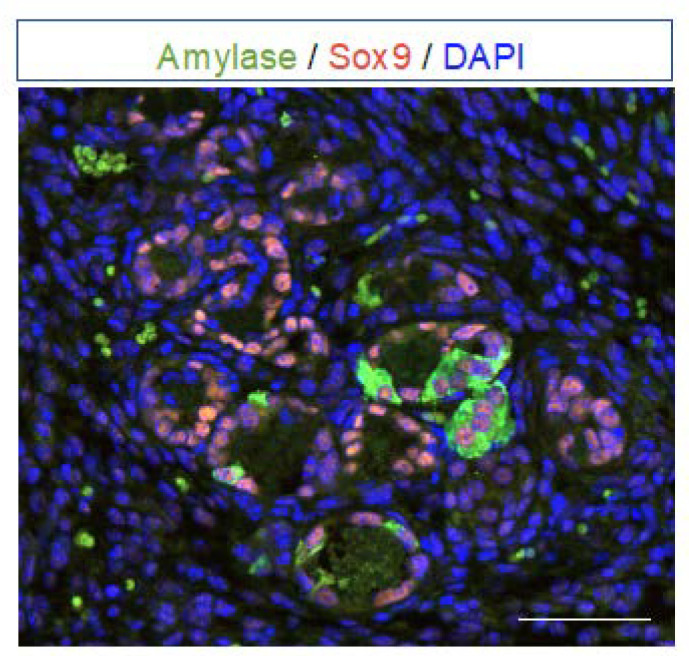
ADM stained with acinar and ductal markers. Amylase (green) and Sox9 (red) immunostainings. Nuclei are stained with DAPI (blue). Forming ADM structures show cells with co-expression of acinar (amylase, cytoplasmic staining) and ductal (Sox9, nuclear staining) markers. Completed ADM structures are stained only by the ductal Sox9 marker. Scale bar: 50 μm. Adapted with permission from Ref. [3], Copyright 2023, Elsevier).

Co-expression of acinar markers and duct markers is used to detect ADM (Figure 2). The co-expression of various digestive enzymes is not completely lost in acinar cells during ADM and there is a concomitant upregulated expression of the duct markers including Sox9 [33], Hnf1b [3,34], Hnf6 [35], Pdx1 [36], CA19–9 [37], CAII [38], CD133 [39], and osteopontin [40] (see Figure 1). 

The transcription factors Hnf1b, Hnf6, Pdx1 and Sox9 are known to play critical roles in the development and differentiation of pancreatic cells [41,42]. Hnf1b is a key member of the transcription factor network implicated in pancreatic MPCs’ control. *Hnf1b* deficiency in embryos leads to pancreas agenesis, showing that Hnf1b is required for pancreas morphogenesis and regional specification of the gut [43]. The sequential activation of Hnf1b, Hnf6 and Pdx1 controls the differentiation of endodermal cells into MPCs [44]. Hnf1b was shown to regulate MPC proliferation, survival and differentiation [45] and was found upregulated in ADM [12,16,23,46,47]. The transcription factor Pdx1 is essential for the specification and differentiation of MPC into endocrine and exocrine cell types. After pancreatic morphogenesis, Pdx1 is required for maintaining the identity and function of mature beta cells. Pdx1 was shown to be up-regulated in human and murine ADM, and persistent expression of Pdx1 in the pancreas causes ADM through Stat3 activation [36]. Sox9 is expressed in pancreatic MPC at E9.5 and is required for proliferation and survival of MPCs [48]. Hnf6 is also expressed in MPCs [49]. In normal adult pancreas, Sox9 and Hnf6 expression is restricted to the duct lineage. *Sox9* and *Hnf6* are up-regulated in human and mouse models of ADM and their overexpression in acinar cells leads to ADM. They are required for repression of acinar genes, for ADM-associated changes in cell polarity and for activation of ductal genes in acinar cells [35]. In order to study to what extent dedifferentiated acini differ from native duct cells and which genes are uniquely regulating acinar cell dedifferentiation, lineage tracing experiments and RNA sequencing were performed with human pancreatic exocrine acinar and duct cells. MECOM, regulated by Sox9, was identified as a transcription factor unique to dedifferentiated acinar cells, critical to maintain cell adhesion and to suppress acinar cell death by permitting cellular dedifferentiation [15].

If the stimuli causing ADM is removed, the acinar tissue is regenerated, and these duct-like progenitor cells formed by ADM proliferate and redifferentiate into acinar cells to replenish the damaged organ. 

## 3. Factors Triggering ADM: Environmental and Cellular Insults

### 3.1. Pancreatitis and Inflammation

Pancreatitis, characterized by inflammation, ADM and fibrosis of the pancreas, can be caused by obstructions such as gallstones migration, by chronic alcohol consumption or by genetic risk factors with mutations occurring in genes encoding acinar digestive enzymes or their inhibitors such as cationic trypsinogen (PRSS1), serine protease inhibitor Kazal type 1 (SPINK1), and carboxypeptidase A1 (CPA1) [50]. These enzymes become inappropriately active in acinar cells or in their immediate microenvironment, leading to autodigestion, cell death and inflammation. Although some acinar cells are lost during acute pancreatitis through necrosis and apoptosis, other acinar cells undergo ADM. Development of pancreatitis is associated with strong up-regulation of inflammatory mediators such as tumor necrosis factor (TNF)α, Interleukin IL-1β, IL-6 and chemokines of the CXC and CC families. IL-1β plays an important role in pancreatitis, as mice overexpressing IL-1β develop ADM [51]. Activation of NF-κB also increases the severity of pancreatitis [52]. NLRP3 inflammasome is activated in pancreatitis notably by reactive oxygen species (ROS) resulting from mitochondrial damage or damage/danger-associated molecular pattern molecules (DAMPs)–cellular components [53]. 

Pancreatitis can be induced experimentally by injection of caerulein into rodents, which induces acinar cell death and inflammation. Caerulein-induced pancreatitis as the injury model of choice in mice has facilitated numerous insights into the cellular and molecular mechanisms of exocrine pancreas regeneration. Caerulein, as an analog of cholecystokinin, directly stimulates the production and secretion of acinar enzymes. Administered at high levels, it causes excessive production of digestive enzymes, leading to acinar cell death and transient ADM, within 1–2 days after caerulein injections. Loss of acinar cell identity in response to caerulein-induced pancreatitis is reversible. ADM are proliferative structures capable of regenerating acinar cells lost in pancreatitis. Remarkably, similar to what occurs in human acute pancreatitis, the pancreas returns to normal morphology, differentiation, and function within 1–2 weeks. Other models such as pancreatic duct ligation, which is a model of obstructive pancreatitis, were also used [54]. 

Obesity and a high-fat diet have been linked to an increased risk of ADM. As an example, obese rats developed ADM, which was reversible after bariatric surgery [55]. The mechanisms generally involved include metabolic dysfunction (insulin resistance and hyperglycemia), oxidative stress (characterized by the generation of reactive oxygen species (ROS) that can damage cellular components) and, most importantly, inflammation, with infiltration of immune cells, particularly macrophages, which secrete cytokines and chemokines [56]. Pro-inflammatory macrophages are activated by a variety of metabolic signals, such as free fatty acids (FFA), lipopolysaccharide (LPS), and glucose. They have been shown to drive ADM by secreting TNFα and the CCL5/RANTES chemokine, factors that activate NF-κB and its matrix metalloproteinases (MMPs) target genes which regulate the extracellular matrix [57]. A recent report also described a novel and intriguing role of cholecystokinin in obesity-associated PDAC. Obesity accelerated PDAC development through changes in the local pancreatic microenvironment driven by increased pancreatic islet (β-cells)-secreted cholecystokinin acting locally on pancreatic acinar cells to accelerate ADM formation [58]. 

Environmental factors such as alcohol or obesity can interfere with the initiation or completion of normal autophagy [59]. Autophagy is a cellular process that plays a critical role in maintaining cellular homeostasis by recycling and degrading intracellular components. Due to their high protein synthetic rates, acinar cells are prone to the accumulation of misfolded proteins. Insufficient autophagy and ER stress contribute to pancreatitis development [59]. Genetically engineered mice models with altered autophagy pathway components have led to chronic pancreatitis with ADM and inflammation, as is the case with ATG5 and ATG7 loss. Impaired autophagy results in ER stress, accumulation of dysfunctional mitochondria and oxidative stress [60,61,62,63]. 

ADM can also be induced by alteration of acinar cell polarity, cell–cell contacts and cell–matrix connections. As an example, loss of Numb, a protein that regulates integrins and cell junctions, accelerates ADM [64]. Additionally, E-cadherin is a key adherence molecule required for maintenance of structural homeostasis. Its stability in epithelial cells is regulated by p120 catenin. Deletion of p120 catenin in pancreatic MPC leads to ADM [65]. Deletion of E-cadherin also leads to ADM, associated with increased inflammation (increased inflammatory cytokines and chemokines, such as Cxcl2, Ccl2, IL-6, TNFα) and fibrosis [66]. 

### 3.2. Signaling Pathways

**Figure 3:**
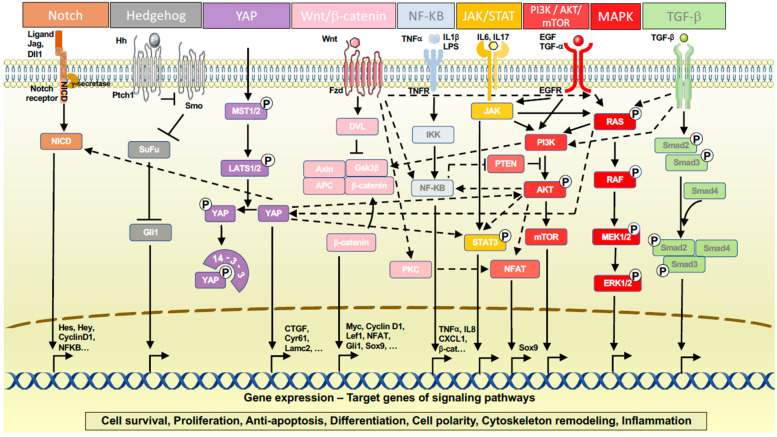
Signaling pathways regulating ADM. Schematic representations of signaling pathways implicated in ADM, namely Notch, Hedgehog, YAP, Wnt/β-catenin, NF-κB, JAK/STAT, EGFR, PI3K/AKT/mTOR, MAPK, and TGF-β, are depicted. Certain interactions between these signaling pathways are indicated. Specific target genes are showcased for some of these signaling pathways.

Several pancreas developmental pathways play important roles in pancreas regeneration and ADM, including Notch, Wnt/β-catenin, Hedgehog, Hippo/YAP, EGFR, MAPK and PI3K/AKT. The ADM process is coordinated by the cross-talk between these signaling cascades. Reactivation of these developmental signaling pathways must be tightly regulated to enable the regeneration of acinar cells while preventing them from persistent ductal reprogramming (Figure 3).

Notch signaling is a key regulator of MPCs maintenance and is required during pancreas embryonic development [67]. Notch intracellular domain (NICD) translocates to the nucleus to activate the expression of target genes, especially *Hes1*. Active Notch signaling has been shown to be required for exocrine regeneration [68]. The loss of Hes1 led to persistent ADM after acute caerulein-induced pancreatitis and impaired regeneration of the exocrine compartment [69]. 

The Hedgehog (Hh) signaling pathway plays a critical role in embryonic development. As a morphogen, Hh signaling prevents pancreas formation, requiring its exclusion for pancreatic bud development [70]. Hh signaling is essential for the efficient regeneration of the exocrine pancreas, as its inhibition leads to impaired redifferentiation resulting in persistent ADM [71,72]. In vertebrates, the primary cilium is the central organelle for the transduction of Hh signaling [73]. While pancreatic ductal cells possess a single primary cilium, acinar cells lack cilia. However, when undergoing ADM, acini assemble a cilium, indicating the need for Hh signaling in the differentiation of progenitor duct-like cells [74].

Primary cilia are cellular sensors that mediate several signaling pathways important for pancreatic development and homeostasis [75]. Genetic deletion of primary cilia in embryonic progenitors leads to cystic ducts, acinar cell apoptosis and ADM. This was shown with *Tg737* mutation (a gene required for cilia formation) [76], *Kif3a* loss (a gene required for cilia construction and maintenance) [77], and *Hnf1b* deficiency (associated with the absence of primary cilia by regulation of target genes such as *Hnf6*, *Pkhd1* and *Kif12*) [45]. In these models, mislocalisation of β-catenin [45,49] and increased cytosolic β-catenin [76] suggested a role for Wnt signaling. Additionally, *Kif3a* mutants expressed high levels of Transforming Growth Factor (TGF)-β ligands and MAPK/ERK pathway activation [77]. 

In post-natal ducts, cilia defects caused by deletion of *Hnf1b*, *Hnf6* or *Lkb1* genes were shown to be associated with enlarged ducts, chronic pancreatitis, acinar apoptosis, ADM, inflammatory infiltrates, fibrosis and adipocyte differentiation [3,78]. Ductal deletion of *Hnf1b* showed enhanced signaling pathways favoring ADM including YAP, Notch, EGFR and TGF-β. This study provided molecular mechanisms by which ciliary defects in ductal cells lead to non-cell autonomous effects on acinar cells notably through the activation of the YAP mechanotransducer [3]. 

Hippo signaling, mediating YAP nuclear localization and downstream activation of target genes, plays an important role in regulating cell proliferation and apoptosis and is crucial to pancreas development and function, including crosstalk with Notch, WNT/β-catenin, and PI3K/AKT/mTOR signaling pathways [79]. Inactivation of Mst1/2 kinases of the Hippo pathway leading to translocation of YAP into the nucleus and activation of target genes induced ADM, immune infiltration and pancreatitis [80]. Dysregulation of the Hippo and PI3K signaling pathways has been observed in human chronic pancreatitis [81,82]. Hippo signaling inactivation associated with YAP activation and dysregulation of PI3K signaling by genetic disruption of *Pten* synergistically promotes ADM through the upregulation of the downstream effector pancreatic connective tissue growth factor (CTGF) [83].

YAP1/TAZ activation in cells undergoing ADM also increases the expression of the JAK–STAT3 pathway components via the upregulation of *Stat3*. JAK–STAT3 signaling is strongly up-regulated in mice with pancreatitis [84]. The JAK/STAT pathway regulates the downstream signaling of numerous membrane proteins and induces the transcription of genes involved in cell proliferation, apoptosis, and inflammatory factor production [85]. YAP1/TAZ signaling has been determined to be necessary and sufficient for ADM induction by regulating JAK-STAT3 signaling [84]. STAT3 is required for ADM formation. P-STAT3 is found in ADM; in addition, loss of STAT3 decreases ADM formation [86]. STAT3 is a critical component of pancreatitis as its deletion protects against pancreatitis. STAT3 supports cell proliferation, metaplasia-associated inflammation, and regulates MMP7 expression [87]. MMP7 was required for ADM in vitro [88], and MMP7-deficient mice were resistant to acinar apoptosis and ADM [89]. IL22 is another activator of STAT3 that promotes ADM [90]. 

Moreover, the YAP/TAZ-STAT3-PYK2-Wnt/β-catenin was shown to constitute a signaling pathway regulating ADM [91]. The Wnt/β-catenin signaling pathway is required in pancreas development [92]. The canonical pathway is activated by Wnt family ligands binding to Frizzled/LRP receptor complexes. The cascade of events that ensues prevents β-catenin degradation within the cytoplasm and allows its stabilization and nuclear translocation. β-catenin signaling was found upregulated during ADM. *β-catenin* null cells were incapable of undergoing ADM, showing a cell-autonomous requirement for intact β-catenin function for acinar cells to undergo ADM [93]. β-catenin was also essential for acinar cell proliferation during regeneration [94,95]. Wnt signaling was thus found to be required for ADM, as well as capable of upregulating MAPK/ERK signaling pathway also promoting ADM [93]. 

Members of the TGF-β superfamily are involved in embryonic development, regulation of homeostasis and diseases [96]. TGF-β signaling occurs through a receptor complex composed of TGF-β receptors which activate the canonical SMAD pathway and non-canonical pathways including MAPK/ERK and PI3K/AKT. In different mouse models, notably by using a dominant-negative type II TGF-β receptor, inhibition of TGF-β reduced caerulein-induced pancreatitis [97,98]. In vitro studies indicate that the activation of TGF-β signaling facilitated ADM of human pancreatic acinar cells, while inhibiting TGF-β signaling reduced the spontaneous ADM of human acinar cells [14,99]. Cell-autonomous expression of a constitutively activated TβRI receptor in acinar cells induced ADM. Activation of TGF-β signaling indeed disrupted acinar cell homeostasis, leading to a drastic increase in the number of ductal structures at the expense of acinar structures, with apoptosis, dedifferentiation of acinar cells and increased expression of transcription factors such as Hnfb1, Sox9 and Hes1. Hnf1b was the best TGF-β responder, and its activation was dependent on the SMAD and MAPK/ERK pathways [47]. 

Epidermal growth factor receptor (EGFR) is a transmembrane glycoprotein, member of the tyrosine kinase family of growth factor receptors. Its extracellular domain provides a ligand-binding site for EGF and TGF-α. The two major intracellular pathways activated by EGFR are the MAPK/ERK pathway and the P13K-AKT pathway. High-level expression of EGFR and its ligands EGF and TGFα were observed in human chronic pancreatitis specimens, as well as the activation of the EGFR signaling pathway in the areas of ADM in mice [100,101,102,103]. Several studies demonstrated that ADM requires EGFR signaling [104,105,106]. *Tgf-α* transgenic mice displayed ADM. Using primary explant cultures and lineage tracing studies, acinar cells were shown to undergo conversion to metaplastic ductal epithelial cells in response to TGF-α and EGFR signaling. Transition of isolated acini to ductal cells in vitro in collagen culture in the presence of TGF-α was comparable to the changes described in TGF-α transgenic mice in vivo. Whereas ADM formation was provoked by transgenic expression of EGFR ligands in vivo, genetic or pharmacological inactivation of EGFR or ligands prevented ADM [101,102]. Among the pathways that integrate EGFR signals, NFATc1/c-Jun was identified as a critical transcription factor complex induced by cell-autonomous EGFR signaling and promoting ADM through Sox9 induction [103]. Moreover, NFATc1 was recently identified as a regulatory hub mediating EGFR signaling, displacement of ARID1, a component of the SWI/SNF chromatin remodeling complex, and induction of ductal gene signature promoting acinar cell reprogramming [107].

Several signaling pathways downstream of EGFR are activated to form a complex regulatory network that coordinately regulate ADM. Kras plays a crucial role in ADM formation and progression [30]. MAPK signaling is tightly regulated and is activated by extracellular growth factor stimulation. After receptor phosphorylation, Kras binds guanosine triphosphate (GTP) and activates the serine/threonine kinase Raf. In turn, Raf phosphorylates and activates MEK1/2, which phosphorylates ERK1/2 that translocates to the nucleus where it promotes transcription and cell cycle progression [108]. Up-regulation of MAPK signaling upon induction of pancreatitis was found in isolated acinar cells [109,110] and in vivo [111]. Activation of MAPK signaling pathway has been demonstrated in human and mouse ADM [112]. ADM from human acinar cells was reduced by MAPK inhibition [12]. Inhibition of MAPK signaling in caerulein-induced pancreatitis in mice by treatment with a MEK inhibitor or by *Mek1/2* knockdown blocked chronic pancreatitis development and reversed caerulein-induced damage, showing that MEK signaling is a driver of ADM required for initiation and maintenance of ADM and limiting organ regeneration [113]. Activation of Kras^G12D^ in adult acinar cells induced ADM in vitro in a 3D collagen matrix and in vivo. Raf/MEK/ERK represents a critical downstream effector pathway through which Kras operates to induce ADM [114,115]. Lineage-tracing studies have confirmed that ADM results from adult acinar cells upon Kras^G12D^ expression [94,114,116]. Kras^G12D^-induced acinar cells had elevated pERK levels [31]. Treatment of cells with a MEK inhibitor efficiently blocked accumulation of pERK, and reduced ADM and MEK1/2 inhibition blocked ADM in the 3D culture [31,112].

*Reg3A* and its mouse homolog Reg3B were up-regulated in human and mouse ADM, respectively, and induced ADM in a 3D culture of primary human and murine acinar cells [117]. Both *Reg3B* transgenic mice and Reg3B-treated mice with caerulein-induced pancreatitis promoted and sustained ADM by activating the MAPK signaling pathway [118]. 

The PI3K/AKT pathway was shown to be involved in ADM [119]. Cellular functions regulated by PI3K signaling include cell transformation, proliferation, growth, motility and survival [120]. Of the four class I PI3K isoforms, a family of heterodimeric lipid kinases that activates downstream kinases such as AKT, only p110α and p110β were expressed in the murine and human pancreas, in particular in the acinar compartment [121]. Constitutive activation of PI3K signaling via the expression of oncogenic p110α^H1047R^ phenocopies Kras^G12D^-induced cellular plasticity by causing ADM through the canonical PI3K/AKT signaling [122]. Pik3ca activating mutation responsible for constitutive activation of PI3K was found to cause increased ADM [123]. Pharmacological selective inhibition of p110α inhibited AKT substrate phosphorylation and completely blocked ADM induced by mutated Kras. Genetic ablation of p110α blocked ADM formation induced by caerulein, whereas the ablation of p110β did not. Moreover, p110α prevented ADM induction by caerulein in a mutated Kras background. The role for p110α in actin rearrangement during ADM was found by activation of the Rho small GTPases that control F-actin network remodeling [121]. Involvement of PI3K activity in ADM was also shown by the pro-tumorigenic effect of restricted loss of the PTEN enzyme, which reverses PI3K activity. Deletion of *Pten* in the pancreas induced an increase in ADM structures [124]. Concomitant conditional *Pten* deletion and Kras^G12D^ activation led to an accelerated and accentuated phenotype of ADM [125]. 

Glycogen synthase kinase-3 (GSK3) β is a serine–threonine kinase involved in several cellular functions by acting as a downstream regulatory switch for numerous signaling pathways, including Wnt/β-catenin and PI3K-AKT signaling pathways. GSK-3β protein expression was elevated following caerulein-induced pancreatitis in mice. GSK-3β promoted ADM in 3D-cultured primary acinar cells and was found necessary for ADM in vivo, contributing to proliferation of ADM cells through activation of the S6 kinase [126,127]. 

The transcription factor Krüppel-like factor 5 (KLF5) has been identified as being up-regulated in ADM by PI3K and MEK signaling. ADM was reduced after genetic inactivation of *Klf5* in vivo through suppression of STAT3 activation. KLF5 is a possible convergence point in MEK and PI3K pathways and a potential link between ductal transformation and increased cellular proliferation during ADM process [127]. Another Krüppel-like factor, KLF4, has an essential role in ADM induction. KLF4 was upregulated and required for ADM; overexpression of KLF4 in acinar cells resulted in ADM [128].

### 3.3. Epigenetic Reprogramming

Epigenetic modifications refer to the chemical or physical changes that affect gene accessibility and expression without altering the DNA sequence. They include (i) DNA methylation, with involvement of DNA methyltransferases (DNMT); (ii) histone modifications (methylation, acetylation, sumoylation, ubiquitination and phosphorylation), notably with histone deacetylases (HDACs), acetylases (HATs) and methyltransferases; (iii) noncoding RNAs [129]. Given the critical role of epigenetic patterns in determining gene accessibility and transcription factor recruitment, it is not surprising that specific epigenetic events can drive acinar cell reprogramming, silencing acinar cell-specific genes and replacing them with the expression of ductal-specific genes.

As an example, HDAC Sirt1 regulates ADM by deacetylating Ptf1a and β-catenin [130]. Metaplastic acinar cells undergo chromatin reprogramming during ADM. Indeed, it has been observed that HDAC activity was up-regulated in the pancreas during pancreatitis [131]. In vivo treatment with the pan-HDAC inhibitors sodium butyrate and trichostatin A (TSA), targeting both class I and class II HDAC subfamilies, reduced ADM, inflammation and fibrosis following induced pancreatitis in rodents [132]. The class I HDAC inhibitor MS-275 modifies the expression of inflammatory molecules and suppresses the migratory properties of macrophages. Moreover, experiments using TGFα-induced acinar transformation suggested that MS-275 prevents acinar dedifferentiation into ADM in a cell-autonomous manner by a down-regulation of the expression of EGFR in acinar cells [131]. TSA was also shown to reverse ADM in mouse and primary human acinar cultures by inhibiting Spink1 and PI3K/AKT signaling [133]. However, in another study, treatment with valproic acid, another pan-HDAC inhibitor, had a detrimental effect on caerulein-induced pancreatitis, manifested by increased inflammation, decreased proliferation and persistent acinar de-differentiation with non-resolving ADM [134]. This highlights the need to further investigate the precise role of HDAC isoforms in the context of ADM. 

Polycomb group (PcG) proteins are histone modifying transcriptional repressors arranged in the Polycomb Repressor Complexes 1 and 2 (PRC1, PRC2). PRC1 contains the BMI1 and the E3 ubiquitin ligase RING1B components which catalyze the mono-ubiquitination of lysine 119 of histone 2A (H2AK119ub). The catalytic subunit of PRC2, EZH2, tri-methylates lysine 27 of histone 3 (H3K27me3). High expression of PRC1 members and enrichment of histone mark H2AK119ub were found in ADM [135,136]. Elevated levels of Bmi1 and Ring1b and their catalyzed histone modification H2AK119ub in ADM were responsible for the epigenetic silencing of acinar transcription factors, such as the complex Ptf1-L containing the DNA-binding subunit Ptf1a and Rbpjl [136]. *Ring1b* knockout mice showed greatly impaired acinar cell dedifferentiation due to a retained expression of acinar differentiation genes [137]. This shows the role of epigenetic repression of acinar-specific differentiation genes as an essential step in ADM. Changes in H3K27Me3 catalyzed by PRC2 also occur and lead to the suppression of genes normally activated by pancreatic injury [138]. *Ezh2* upregulation occurs after pancreatic injury and conditional inactivation of *Ezh2* in mouse acinar cells, leading to defective regenerative response and resulting in compromised proliferation and persistent metaplastic lesions. Ezh2 is crucial for maintaining gene silencing of the p16INK4A cell cycle inhibitor in ADM during acinar cell regeneration [139]. 

BRG1 is a component of the SWI/SNF chromatin remodeling complexes. Deletion of Brg1 in acinar cell mice drastically attenuated the formation of ADM, and *Sox9* expression was downregulated in the BRG1-depleted ADM. BRG1 bound to the *Sox9* promoter to regulate its expression and was critical for recruitment of upstream regulators, including PDX1, through a local change in chromatin conformation in acinar cells [140]. Genome-wide analysis (ChIP-Seq and ATAC-Seq) in acinar cells revealed that another SWI/SNF subunit, ARID1A, remodels chromatin architecture and stabilizes acinar cell identity. Loss of Arid1a decreases chromatin accessibility of genes that produce digestive enzymes during ADM [141]. 

miRNA can also regulate ADM. miR-802 is a highly abundant and acinar-enriched pancreatic miRNA which suppresses ADM. Genetic ablation of mir-802 facilitated ADM in caerulein or *Kras*^G12D^-induced ADM. miR-802 deficiency resulted in de-repression of a miR-802 target network, leading to the activation of F-actin rearrangement and *Sox9* expression [142]. 

## 4. Progression of ADM to Pancreatic Intraepithelial Neoplasia (PanIN)

The relevance of ADM to pancreatic ductal adenocarcinoma (PDAC) is supported by the observation that ADM is frequently associated with human PanIN lesions in PDAC patients [115,143]. Chronic pancreatitis is a well-known risk factor for PDAC development in humans. Patients with hereditary pancreatitis showed an increase in pancreatic cancer incidence [144]. In patients with pancreatitis, ADM is transient and reversible. However, persistent ADM can progress to PanIN, and finally to PDAC. The progression from ADM to PanIN has been well established both in mouse models by lineage tracing [114] and in humans [30,125,145]. It has been observed that ADM lesions are correlated with the invasive front of pancreatic cancer, contributing to desmoplasia and cancer cell invasion of the local parenchyma [146]. Mouse models, supported by human histopathological studies, demonstrated that PanIN lesions are the precursor for PDAC and often already exhibit mutations characteristic of PDAC [147,148].

The progression of ADM to PanIN is promoted by the factors that induce ductal cell identity such as Sox9, but suppressed by the factors that preserve acinar cell properties such as Ptf1a and Mist1 [25,30,128,149]. Both morphology and gene expression changes are consistent with the connection between ADM and PanIN. *Ptf1a* is epigenetically silenced during inflammation and oncogenic Kras-driven ADM. *Ptf1a*-deficient acinar cells are dramatically sensitized to Kras transformation, and loss of *Ptf1a* accelerates the development of invasive PDAC [25]. Forced expression of the acinar-restricted transcription factor Mist1 significantly attenuates Kras^G12D^-induced ADM/PanIN formation, showing that maintaining Mist1 activity in Kras^G12D^ expressing acinar cells can partially mitigate the transformation activity of oncogenic Kras [31]. Loss of Nr5a2 cooperates with oncogenic Kras to drive ADM and PanIN development [32]. Reactivation of progenitor functions supports tumorigenicity. Transcription factors that are up-regulated in ADM, such as Sox9, Hnf6, Pdx1 and Hes1, are also up-regulated in PanIN. Loss of *Hnf6* expression has been shown to correlate with human pancreatic cancer progression [150]. Sox9 stimulates gene expression that leads to ADM, PanIN and initiation of PDAC in mice [149]. Sox9 expression in patient tumor samples is elevated at all stages of PanIN lesions and PDAC, correlating with the increased expression of EGFR pathway-related genes. Similarly, in mice, the absence of Sox9 reduces EGFR signaling and pancreatic tumorigenesis [151]. Elimination of Brg1 in acinar cells impairs the formation of Kras^G12D^-induced ADM and PanIN, associated with the downregulation of *Sox9* expression in *Brg1*-depleted ADMs/PanINs, showing that Brg1 is critical for PanIN initiation and progression through positive regulation of *Sox9* [140]. 

Impaired acinar cell differentiation favors the development of PanIN in the context of oncogenic signals. Oncogenic Kras mutation represents the most frequent and earliest genetic alteration in PDAC patients, highlighting its role as a driver of PDAC [152]. The cellular plasticity in ADM is the key to pancreas regeneration as well as tumorigenesis. In the context of a physiological response to acute pancreatic insult, ADM is a reversible process with the capacity of the metaplastic cells to proliferate and replenish the damage organ. However, in the case of persistent aberrant growth factor signaling or concomitant oncogenic activation such as the Kras^G12D^ activating mutation, the metaplastic cells cannot revert to a differentiated state, leading to irreversible ADM, facilitating the onset of PanIN and PDAC development. ADM development has been shown to precede PanIN formation in mouse Kras^G12D^ models [115]. Kras^G12D^-expressing acinar cells in the 3D culture rapidly convert to ductal cells that mimic the properties associated with in vivo ADM and PanIN lesions [31]. Oncogenic *Kras* mutation compromises the ability of acinar cells to regenerate following acute pancreatitis and locks metaplastic cells in a persistently ADM state that can rapidly give rise to PanIN and promote PDAC development [94,153]. Kras^G12D^ expression in adult acinar cells generates ADM lesions that progress to PanIN and PDAC [114,147,154,155]. Lineage-tracing experiments in mice have demonstrated that PanIN lesions are mainly derived from acinar cells undergoing ADM [149]. ADM is thus the earliest pre-neoplastic lesion that predisposes to PDAC, making ADM reprogramming a crucial step in pancreatic cancer initiation.

The MAPK pathway hijacks the plasticity of acinar cells to promote tumorigenesis. MAPK signaling is a key Kras effector for PanIN maintenance. MAPK activity is sufficient and required for ADM and PanIN formation [112]. MEK1/2 inhibition results in PanIN regression in Kras-activated GEMM. The MAPK pathway is required for maintaining PanIN lesions by promoting acinar dedifferentiation. Concomitant conditional *Pten* deletion and Kras^G12D^ activation accelerate and accentuate the phenotype of ADM, PanIN, and malignant progression. Concurrent dysregulation of the PTEN/PI3K/AKT and MAPK pathways acts synergistically to promote PanIN initiation and progression [125]. A strong activation of PI3K signaling was observed in ADM and in PanIN [122]. The requirement for PI3K p110α in acinar cell plasticity and the initiation of cancer induced by oncogenic Kras has been established [121]. In mutant Kras-driven mouse models, the activation of the EGFR signaling pathway in the areas of ADM and PanIN was convincingly pronounced, with downstream ERK activation. EGFR or ligand inactivation using either genetic or pharmacological approaches preserves acinar cells in a well- differentiated state and almost completely abolishes ADM and PanIN induced by Kras^G12D^ and inflammation [101,102].

Mutant Kras, injury, and stress signaling converge to activate KLF4 expression. KLF4 synergizes with mutant Kras in PanIN initiation. Overexpression of KLF4 results in sustained ADM, which promotes the initiation of PanIN in the presence of mutant Kras [128]. 

Important signaling pathways for pancreatic development, including Notch signaling, are involved in ADM progression to PanIN [156]. Notch signaling is also involved in tumor initiating processes, development of ADM and PanIN lesions, as well as in tumor progression [116,156]. Hes1 was shown to be required for Kras^G12D^-driven PanIN formation [157]. Metaplastic acinar structures notably express Notch target genes, EGFR, ErbB2 and pErk. This expression pattern parallels the expression pattern detected in PanIN, suggesting that ADM and PanIN follow similar molecular pathways [115].

Other signals contribute to the initiation or progression of ADM lesions to PanIN. They include IL6 secretion combined with downstream STAT3 activation [86,87]. REG3β expression is induced during ADM and in early PanIN by IL17, and REG3β-promoted PanIN development induced by the Kras^G12D^ oncogene through the GP130/JAK2/STAT3 pathway [158]. Pro-inflammatory macrophages also play a critical role in ADM to PanIN transition. Depletion of macrophages in mice expressing oncogenic Kras under an acinar cell-specific promoter results in a decreased progression of ADM to PanIN [159]. The transcriptional regulators YAP/TAZ are required for Kras^G12D^ to promote PanIN. In the absence of YAP, Kras^G12D^ is unable to induce PanIN lesions, suggesting that acinar-to-ductal reprogramming via YAP/TAZ is a mandatory step in pancreatic cancer initiation from acinar cells [84]. Activation of TGF-β signaling in combination with oncogenic Kras activation leads to the early onset of PanIN that can naturally evolve toward high-grade/locally invasive lesions [47]. 

Several molecular alterations occur during the progression from ADM to PanIN, including mutations in tumor suppressor genes such as CDKN2A (also known as p16/INK4a), p53 [160] and the cyclin-dependent kinase inhibitor p21 [161], thus regulating cell cycle, genome stability, apoptosis and DNA repair. Recently, a “super-tumor suppressor” P53 mutant was found to inhibit ADM and PanIN proliferation driven by Kras^G12D^ [162].

Altered chromatin remodeling can foster ADM to PanIN progression. Chromatin reshaping occurs in metaplastic cells and synergizes with master transcriptional regulators to render ADM irreversible. *Ezh2* deficiency accelerated PanIN progression [139]. Loss of Arid1a, member of the SWI/SNF complex, counteracts acinar cell identity and cooperates with oncogenic Kras in driving pancreatic carcinogenesis [141]. As another epigenetic regulation, acinar cell-specific genetic ablation of miR-802 significantly increased the number of ADM and PanIN lesions and reduced the median survival compared with control mice [142].

Thus, ADM is a pre-neoplastic event critical for eventual malignant transformation. While development of ADM consists in an adaptative stage to environmental stress, the progression of metaplasia can be considered as an oncogenic stage. A recent study identified populations in human samples associated with ADM using single-cell RNA sequencing (scRNA-seq), and lineage trajectory analyses predicted that some ADM populations were related to PanIN, showing that the sequence ADM-PanIN-PDAC identified in mouse models can accurately reflect tumor progression in human patients [163]. Deciphering the mechanisms of metaplasia formation is important for understanding tissue homeostasis and adaptation to stress, and studying the progression of metaplasia to PanIN neoplasia may allow identification of important factors that contribute to early-stage malignancy.

## 5. Heterogeneity of Metaplastic Cells Revealed by scRNA-seq

Recently, scRNA-seq analyses revealed a higher epithelial heterogeneity in ADM than previously appreciated (see Figure 1). Moreover, changes between injury-induced ADM and Kras^G12D^ oncogene-induced ADM were identified. Metaplastic cells appear to be not a homogenous population but rather can be divided into distinct metaplastic lineages that infiltrate pancreatic lesions. A recent study identified Onecut2 and Foxq1, specifically and highly expressed in metaplastic cells. Onecut2 was reported to have a major role in prostate cancer, and Foxq1 is a transcription factor that regulates *Muc5ac*. Their expression may be needed to regulate the transition from early metaplastic stage to late metaplastic stage. Moreover, metaplastic cells include several subpopulations with distinct transcription programs. Metaplastic cell clusters include transcription signatures of tuft-like cells (Dclk1 positive), stomach pit-like cells (Muc5ac positive), stomach chief cells, neuroendocrine-like cells and senescent cells [164]. The formation of a significant population of chemosensory tuft cells with a pancreatobiliary identity (expressing the markers Dclk1, Pou2f3, and Trpm5) was previously identified during ADM [165,166,167]. While tuft cells were rarely found in caerulein-induced transient ADM, they were found to represent 15% of ADM in TGFα- or Kras^G12D^-induced ADM and were also present in PanINs [165,167]. Dclk1-positive cells were characterized as a subpopulation with cancer stem cell properties [165,167,168,169,170]. Another scRNA-seq study of murine and human ADM revealed that a mucin/ductal population resembling gastric pyloric metaplasia can generate tuft and enteroendocrine cells. Then, the activation of Kras^G12D^ in an injury-induced Hnf1b+ ADM led to neoplastic transformation and the formation of Muc5ac-positive gastric-pit-like cells [171]. Kras^G12D^ expression was found to drive PanIN-specific changes in ADM, characterized by significantly higher expression of proto-oncogenes such as Fosl1 and Junb, known downstream targets of Kras^G12D^. Answering the question of which metaplastic cells within a lesion undergo malignant transformation is crucial to understanding the development of PDAC, as the specific cellular state at the time of acquiring an oncogenic mutation could be involved. Identifying this sequence of events can provide valuable insight into the origin of malignant cells and the onset of PDAC.

## 6. Conclusions

PDAC is a devastating disease with poor survival rates. Studies have pointed to an acinar cell origin in which acinar cells undergo ductal metaplasia at the onset of tumor formation. ADM or, as a more appropriate terminology, acinar cell reprogramming, is a mechanism needed for regeneration after inflammation or injury. It results from acinar cell identity marker silencing and activation of acinar cell dedifferentiation drivers. However, in the presence of oncogenic signaling, ADM is irreversible and leads to PanIN. Future research and clinical translation should continue to focus on using appropriate disease models, identifying suitable ADM detection markers, as well as discovering molecular targets that can impede the initiation and progression of ADM and enhance regeneration following pancreatic injury. Understanding the intermediate states of ADM and important molecules that regulate ADM formation may help the development of novel preventive strategies that could be translated to the clinical setting. The validation of whether manipulating molecular targets can hinder the formation of PanIN from ADM and using these markers for early detection of PDAC remains to be conducted preclinically and clinically. Since PanIN lesions are the most common precursor lesions and are currently clinically undetectable, understanding the mechanisms underlying formation and progression of premalignant lesions is critical for early detection and therapeutic intervention of PDAC.

## Data Availability

Not applicable.
